# Methicillin-Sensitive Staphylococcus aureus-Associated Leukocytoclastic Vasculitis: A Case Report and Literature Review

**DOI:** 10.7759/cureus.60867

**Published:** 2024-05-22

**Authors:** Dinara Salimova, Marwah Alchalabi, Bekure B Siraw, Shayet Hossain Eshan, Monica Sharma

**Affiliations:** 1 Internal Medicine, Ascension Saint Joseph Hospital, Chicago, USA; 2 Infectious Disease, Ascension Saint Joseph Hospital, Chicago, USA

**Keywords:** surgical site infection, petechial rash, leukocytoclastic vasculitis, vasculitic rash, methicillin-sensitive staphylococcus aureus

## Abstract

The association of* Staphylococcus aureu*s with vasculitis remains relatively rare and poorly understood. In this report, we present a case of Methicillin-sensitive *Staphylococcus aureus* (MSSA)-associated leukocytoclastic vasculitis (LCV) following a surgical site infection, adding to the limited body of knowledge on this intriguing clinical entity.

A 52-year-old male with a medical history significant for type 2 diabetes mellitus, hypertension, hyperlipidemia, and coronary artery disease presented with progressively worsening generalized petechial rash and migratory joint pains with associated joint swelling. The patient's symptoms began following surgical repair for a rectus abdominis incisional hernia with mesh placement that was complicated by an abdominal wall abscess at the surgical site, prompting drain placement. Cultures from the abscess aspirate revealed Methicillin-sensitive *Staphylococcus aureus* infection. A punch biopsy of the petechial lesions revealed findings consistent with leukocytoclastic vasculitis. The rash and joint pains resolved approximately one week after initiation of treatment with antibiotics and steroids.

This case sheds light on the rare but clinically significant association between Methicillin-sensitive *Staphylococcus aureus* infection and leukocytoclastic vasculitis, particularly following surgical site infections. The prompt recognition and treatment of underlying MSSA infection, along with the targeted management of LCV, resulted in the resolution of symptoms in our patient. This case emphasizes the importance of a comprehensive diagnostic approach and highlights the efficacy of antibiotic therapy in mitigating MSSA-associated vasculitic manifestations.

## Introduction

*Staphylococcus aureus* (*S. aureus*) is a well-recognized pathogen capable of eliciting a wide array of clinical manifestations, ranging from septic shock to suppurative infections [1]. Beyond its direct pathogenic effects, *S. aureus* is also known to provoke immune system activation, resulting in various clinical presentations, including toxic shock syndrome associated with superantigenicity and hypersensitivity [2]. While the bacterium's propensity for causing endovascular complications, such as infective endocarditis and vascular graft infection, is extensively documented, its association with vasculitis remains relatively rare and less understood. Specifically, the literature on vasculitis associated with *S. aureus* infection, particularly Methicillin-sensitive *Staphylococcus aureus* (MSSA), is sparse, with only a few reported cases documented. In this report, we present a case of MSSA-associated leukocytoclastic vasculitis (LCV) following a surgical site infection, adding to the limited body of knowledge on this intriguing clinical entity.

## Case presentation

A 52-year-old male with a medical history significant for type 2 diabetes mellitus, hypertension, hyperlipidemia, and coronary artery disease presented with complaints of fatigue, weakness, progressively worsening generalized rash, and migratory joint pains with associated joint swelling.

About four weeks before presenting to our clinic, the patient underwent surgical repair for a rectus abdominis incisional hernia with mesh placement. Subsequently, he developed an abdominal wall abscess at the surgical site, prompting drain placement. About a week before drain placement, the patient started experiencing migratory joint pains and swelling. On the same day as the drain placement, he developed a rash on his left foot, subsequently spreading gradually to involve bilateral feet, legs, thighs, hands (including palms), forearms, arms, abdomen, and back. Cultures from the abscess aspirate revealed Methicillin-sensitive *Staphylococcus aureus* infection. Upon the onset of the rash, the patient was initially admitted to another hospital, where he received treatment with Augmentin and Meropenem. The aforementioned antibiotics were started the following day after the onset of the rash and discontinued upon admission to our facility.

At the time of admission to our facility, his home medication regimen included aspirin, clopidogrel, atorvastatin, carvedilol, empagliflozin, metformin, tirzepatide, lisinopril, and gabapentin. He complained of progressively worsening rash, joint pains, fatigue, and malaise. He was afebrile, not tachycardic, with systolic blood pressure ranging between 120 and 140 mmHg, diastolic blood pressure ranging between 80 and 90 mmHg, normal respiratory rate, and normal oxygen saturation on room air.

**Figure 1:**
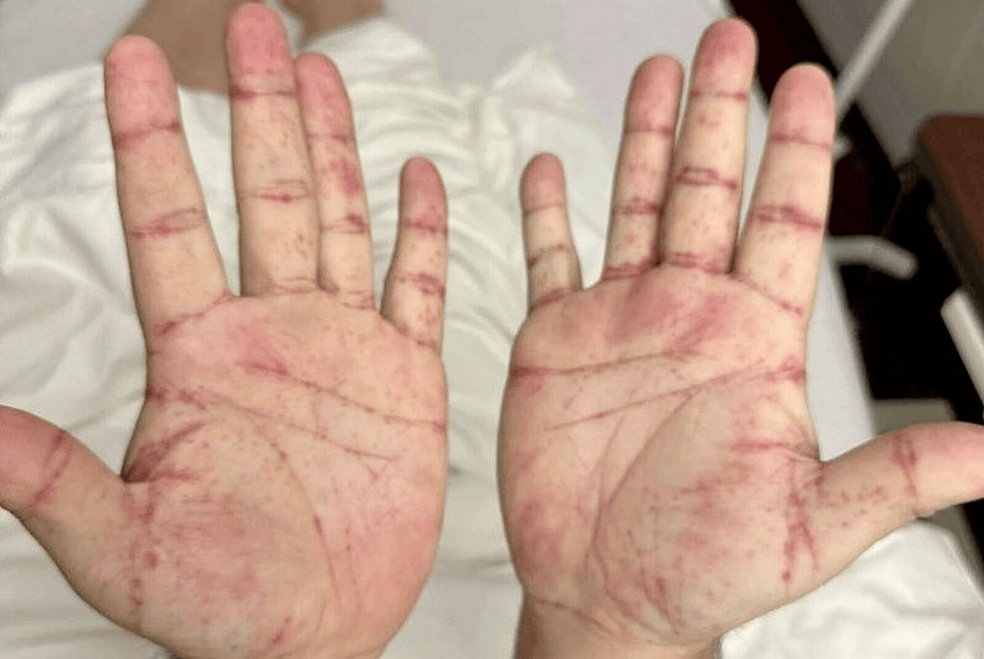
Non-tender, non-palpable petechiae distributed over bilateral palms

**Figure 2:**
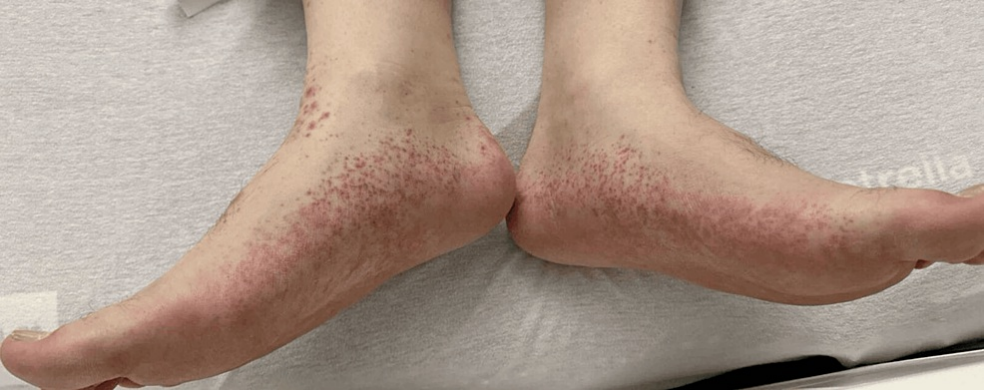
Non-tender non-palpable petechiae distributed over bilateral feet

Physical examination revealed non-tender, non-palpable petechiae distributed over bilateral feet (including soles), bilateral legs, thighs, abdomen, back, bilateral arms, forearms, and hands (including palms) (Figures 1, 2). The face and neck were spared. Generalized joint swelling and tenderness were observed involving the ankles, knees, elbows, wrists, and small joints of the hands symmetrically. Tenderness was noted in the epigastric area at the site of mesh placement and drain location, with a functional accordion drain present. Abdominal examination was otherwise unremarkable.

**Figure 3:**
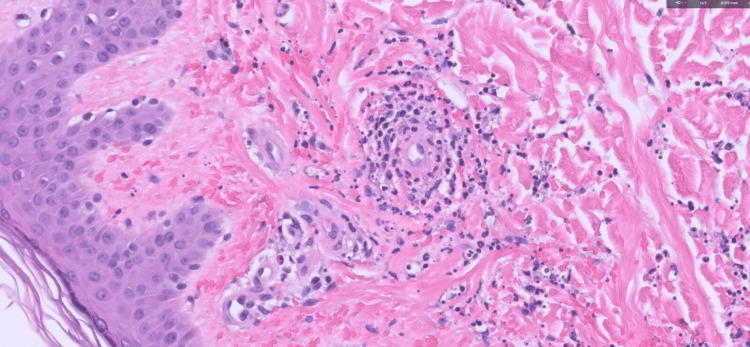
Hematoxylin and eosin staining, magnification 200x Within the papillary and reticular dermis are blood vessels surrounded by acute inflammatory cells with focal fibrinoid necrosis of small vessel walls. Fibrin deposition is noted. Karyorrhectic debris is seen. The findings are consistent with leukocytoclastic vasculitis.

Extensive blood work revealed leukocytosis (15.3 cells/mm^3^) with elevated absolute neutrophil count (72.3%), elevated C-reactive protein (CRP) levels (4.7 mg/L), positive antinuclear antibody (ANA), with mild elevation in anti-extractable nuclear antigen (anti-ENA) and ribonucleoprotein antibody (4.3 units/mL). The anti-neutrophil cytoplasmic antibody (ANCA) panel was negative, as were hemolysis and coagulation workup, syphilis test, human immunodeficiency virus (HIV), and hepatitis serologies. Serum immunoglobulin levels and complement C3 and C4 levels were within normal limits. Urinalysis and renal function tests were normal upon admission. Transesophageal echocardiography ruled out infective endocarditis. Unfortunately, blood culture results were not obtained, given the patient's ongoing antibiotic treatment at the time of admission to our facility. A punch biopsy of the petechial lesions from the right foot revealed acute inflammatory infiltrate around blood vessels with fibrinoid necrosis, and karyorrhectic debris consistent with leukocytoclastic vasculitis (Figure 3).

The patient was initiated on intravenous Cefazolin and steroids (intravenous methylprednisolone followed by oral prednisone), with a trial of colchicine, which was discontinued due to diarrhea. The positive ANA and antinuclear ribonucleoprotein (anti-RNP) antibodies were considered to be false positives in the context of vasculitis and were attributed to the patient's underlying connective tissue disease (CTD). The rash and joint pains resolved approximately one week after the initiation of treatment, although joint swelling persisted for about a week but gradually improved. Follow-up evaluations were planned with Rheumatology for ongoing management of his underlying connective tissue disease.

## Discussion

Leukocytoclastic vasculitis represents a distinct form of inflammation affecting small vessels, characterized histologically by neutrophilic infiltration within and around vessel walls, fibrinoid necrosis, and signs of tissue damage [3]. The incidence of LCV ranges from 15 to 38 cases per million/year, with a prevalence of 2.7 to 29.7 per million [4]. Four major etiologic groups of LCV have been identified, including antineutrophilic cytoplasmic antibody (ANCA)-associated, immune-complex-associated vasculitis associated with systemic diseases (such as rheumatoid arthritis, systemic lupus erythematosus, and sarcoidosis), and vasculitis associated with probable etiology (drugs, infections, sepsis, and neoplasms) [5]. In up to 50% of cases, LCV is idiopathic [6]. Post-infectious LCV most commonly follows streptococcal upper respiratory tract infections, although other infectious triggers have been documented, including *Staphylococcus aureus, Mycobacterium, Chlamydia, Neisseria, HIV, Syphilis, *and *Hepatitis C and B* [6].

The exact pathogenesis of MSSA-associated LCV remains unclear; however, it is generally believed to involve immune complex deposition in vessel walls and activation of the complement system, leading to neutrophil chemotaxis and subsequent vessel wall injury [6,7]. *S. aureus* has superantigens and other antigenic factors that may stimulate antibody production, contributing to immune complex formation and complement activation [2,8].

Clinically, cutaneous LCV typically presents as symmetrically distributed, non-blanchable petechiae and palpable purpura, often affecting the lower extremities [4]. Fever, malaise, and arthralgias may accompany cutaneous manifestations [6]. Our patient presented with classical clinical features of LCV following drainage of an MSSA-associated abdominal wall abscess, suggesting a potential etiologic association between the infection and vasculitis. Although the lack of verified bacteremia poses a limitation, the temporal relationship between abscess intervention and rash development, as well as symptom resolution with antibiotic treatment, supports this association. 

Two analogous case reports were identified in the literature. Lokineni et al. documented a case of Methicillin-sensitive *Staphylococcus aureus *(MSSA) osteomyelitis presenting with a non-healing leg ulcer, exhibiting histopathological features indicative of leukocytoclastic vasculitis (LCV), and achieving ulcer resolution subsequent to osteomyelitis treatment [9]. Similarly, Mosher et al. presented a case of MSSA bacteremia associated with palpable purpura and positive perinuclear antineutrophil cytoplasmic antibody (p-ANCA), where symptom alleviation followed bacteremia treatment with antibiotics [10]. Although our patient had more systemic involvements and his skin lesions were different, all three patients improved significantly with treatment of their *S. aureus *infections supporting the likely association between the *S. aureus* infection and LCV. 

Diagnosis of LCV relies on skin biopsy for histopathologic evaluation and immunofluorescence studies [4]. Although the latter was not performed in our case, the former was consistent with LCV. Laboratory workup is essential to exclude systemic causes, including complement levels, CRP, ANCA, ANA, and complete blood count [4]. Control of the infectious source is paramount in managing LCV associated with infection. Despite the possibility of drug-induced vasculitis, the resolution of the rash with cefazolin therapy in our case suggests MSSA eradication as the primary mechanism. Adjunctive therapies such as corticosteroids, colchicine, and dapsone may be considered for refractory cases [4,6].

## Conclusions

This case sheds light on the rare but clinically significant association between Methicillin-sensitive* Staphylococcus aureus* (MSSA) infection and leukocytoclastic vasculitis (LCV), particularly following surgical site infections. The prompt recognition and treatment of underlying MSSA infection, along with the targeted management of LCV, resulted in the resolution of symptoms in our patient. Moreover, the temporal correlation between abscess intervention and rash onset, coupled with analogous case reports, underscores the potential causative role of* S. aureus* in LCV pathogenesis. This case emphasizes the importance of a comprehensive diagnostic approach and highlights the efficacy of antibiotic therapy in mitigating MSSA-associated vasculitic manifestations.
